# The Shuganhuazheng Formula in Triple-Negative Breast Cancer: A Study Based on Network Pharmacology and In Vivo Experiments

**DOI:** 10.1155/2020/8173147

**Published:** 2020-12-14

**Authors:** Bo Wang, Rui Fei, Yan Yang, Niancai Jing, Yi Lu, Hongyu Xiao, Jili Yang, Yue Zhang

**Affiliations:** ^1^Changchun University of Chinese Medicine, Changchun, Jilin 130117, China; ^2^Cell Biology Department, School of Basic Medicine, Jilin University, Changchun, Jilin 130021, China; ^3^Department of Integrated Chinese and Western Medicine, Jilin Cancer Hospital, Changchun, Jilin 130000, China

## Abstract

Breast cancer is the most common cancer in women. Among breast cancer subtypes, triple-negative breast cancer (TNBC) has the highest degree of malignancy and the worst prognosis. The Shuganhuazheng formula (SGHZF) is a traditional Chinese herbal formula for the treatment of TNBC, but the mechanism of SGHZF in the treatment of TNBC remains unclear. In this study, the therapeutic effect and mechanism of SGHZF against TNBC were preliminarily determined based on in vivo experimental verification and network pharmacology. In terms of therapeutic effects, the antitumour effect was verified by measuring and calculating tumour volume, and the expression of proto-oncogene c-Myc was verified by PCR. In terms of the mechanism, potential therapeutic targets were identified by overlapping the SGHZF-related and TNBC-related targets. After comprehensively analysing the results of the protein-protein interaction (PPI), gene ontology (GO) function, and Kyoto Encyclopedia of Genes and Genomes (KEGG) pathway enrichment analyses, Akt and HIF-1*α* were selected for verification by using immunohistochemical and Western blot analyses. The results of the study indicated that SGHZF can inhibit breast tumour growth in mice and that the mechanism may be related to the inhibition of Akt and HIF-1*α* expression.

## 1. Introduction

Malignant tumour disease has become a major threat to human life and health. According to the Global Cancer Statistics of the World Health Organization (WHO) in 2018, 1/10 women will suffer from cancer in her lifetime; breast cancer is the most common cancer in women, accounting for 24.2% of female cancer cases. Breast cancer is also the leading cause of cancer-related death in women, accounting for 15% of female cancer-related deaths [1]. Triple-negative breast cancer (TNBC) accounts for 10–20% of all breast cancers [2]. TNBC is highly invasive, has a poor prognosis, and is prone to local recurrence and distant metastasis [3, 4]. At present, chemotherapy is still the main treatment for TNBC, but several unavoidable side effects are associated with this strategy [5, 6]. Therefore, people have searched for effective complementary and alternative medical methods for a long time [7]. As an important type of complementary and alternative medicine, traditional Chinese medicine (TCM), has been widely used in the treatment of TNBC in China and many other countries and regions of the world with significant curative effects and few side effects [8, 9].

The Shuganhuazheng formula (SGHZF) is an empirical formula for the treatment of TNBC that was created by Professor Zhang Yue, a famous expert in TCM in Jilin Province. It consists of Chai Hu, Bie Jia, Dang Gui, Bai Zhu, Bai Shao, Zhe Bei, Fu Ling, and Gan Cao. It has been widely used in the Department of Integrated Traditional Chinese and Western Medicine of Jilin Cancer Hospital for many years. However, the mechanism by which SGHZF helps to treat TNBC remains unclear, and this lack of understanding affects its potential clinical application to some degree. In this study, the therapeutic effect and mechanism of SGHZF against TNBC was preliminarily determined based on network pharmacology and in vivo experimental verification. The purpose of this study is to provide an important experimental basis for the clinical application of SGHZF in the treatment of TNBC. The specific research process is as follows.

## 2. Materials and Methods

### 2.1. Drugs, Cells, Animals, and Reagents

SGHZF patent medicine was provided by the Jilin Institute of TCM (1 g is equivalent to 5.3125 g of crude drugs), and it was prepared at different concentrations as needed for the experiments. The 4T1 cells were purchased from the Shanghai Cell Bank of the Chinese Academy of Sciences. Female BALB/c mice were purchased from Changchun Yisi Experimental Animal Technology company with limited liability (SCXK (Ji)-2016-0003). c-Myc, Akt, and HIF-1*α* antibodies were purchased from Abcam. Relevant primers were purchased from Yugong Bioengineering (Shanghai) Co., Ltd. Other reagents were purchased from (Changchun) Fengsheng Biological Co., Ltd.

### 2.2. Establishment of Tumour-Bearing Mice with TNBC and Determination of the Antitumour Effect of SGHZF

All animal experiments were conducted under the guidelines of the Animal Care and Use Committee of Changchun University of Chinese Medicine. After 1 week of adaptive feeding, the mice were fasted in a barrier environment at room temperature for 12 hours. Forty female BALB/c mice weighing 18 g–22 g and aged 8 weeks were selected. After anaesthesia by intraperitoneal injection of 2% pentobarbital at doses of 3–4 *μ*l/g, 4T1 cells (3 × 10^5^ cells/ml) were inoculated into the anterior breast of their axillary sternum, and each mouse was injected with 0.1 mL. On the 3^rd^ day, tumour nodules with diameters of 3 mm–4 mm were observed at the inoculation site, which was considered successful modelling [10]. On the second day after successful modelling, 40 mice (8 per group) were randomly divided into a model control group and SGHZF experimental groups receiving 0.01 g/kg, 0.1 g/kg, 1 g/kg, and 10 g/kg doses. Each experimental group was administered 0.2 ml of the corresponding concentration of SGHZF by intragastric administration, while the model control group was administered the same amount of distilled water by intragastric administration once a day for 21 days. The longest and shortest diameters of the tumours were measured on days 3, 7, 14, and 21 to calculate the tumour volume and tumour inhibition rate. The formula for calculating the tumour volume was as follows: tumour volume = (longest tumour diameter × shortest tumour diameter^2^)/2. The formula for calculating the tumour inhibition rate was as follows: tumour inhibition rate = (average volume of tumours in the model control group − average volume of tumours in each experimental group)/average volume of tumours in the model control group ^*∗*^ 100%.

### 2.3. Immunohistochemical Analysis

The mice were treated via the method described in section 2.2. On the 21^st^ day, the mice were killed by asphyxiation, and the tumour tissues were dissected. Then, tumour tissues were fixed in 10% neutral formalin solution according to routine tissue treatment protocols. After paraffin embedding, serial sections were made (2 *µ*m thick). Finally, the tissue morphology was observed by haematoxylin and eosin staining (H&E) under an electron microscope. Immunohistochemical analysis was performed by staining the sections using different antibodies (according to the antibody instructions). Motic Images Advanced 3.2 was used as the image analysis software.

### 2.4. PCR

The mice were treated via the method described in section 2.2. On the 21^st^ day, the mice were killed by air asphyxia to dissect the tumour tissues. After the tumours were harvested from the mice, they were fully ground in liquid nitrogen, total RNA was extracted strictly following the kit instructions, and PCR amplification was followed by 1.5% agarose gel electrophoresis. Finally, the greyscale values of the bands were analysed by ImageJ software.

### 2.5. Western Blot Detection

The mice were treated with the method described in section 2.2. On the 21^st^ day, the mice were killed by air asphyxia to dissect the tumour tissues. Then, protein extraction and quantitative analysis were performed on tumour tissues. After routine detection of protein concentration and denaturation of proteins, SDS-PAGE electrophoresis and membrane transfer were carried out. Finally, the grey values were analysed by ImageJ software.

### 2.6. Data Preparation of Network Pharmacology Research

TCMSP [11] (http://lsp.nwu.edu.cn/tcmsp.php) and TCMID [12] (http://119.3.41.228:8000/tcmid) databases were used to search for the active components of SGHZF. The search terms were “Chai Hu”, “Bai Shao”, “Bai Zhu”, “Chuan Bei”, “Dang Gui”, “Fu Ling”, and “Gan Cao”. Then, the TCMSP database was used to screen the active components and retrieve their relevant targets. The screening conditions were oral bioavailability (OB) ≥30%, drug-likeness (DL) ≥0.18, and Caco-2 permeability >−0.4. Finally, the retrieval results were combined and duplicates were removed to obtain the targets of the active components of SGHZF. Because turtle shells are not suitable for network pharmacology research, we decided to exclude them.

The GENECARDS [13] (https://www.genecards.org/) and OMIM [14] (https://www.omim.org/) disease databases were used to search disease targets of TNBC. The search term was “triple-negative breast cancer.” Finally, the retrieval results were combined and duplicates were removed to obtain the targets of TNBC.

### 2.7. Construction of Network Graph and Potential Therapeutic Targets Analysis

R language software was used to analyse the interaction between drugs and disease targets to obtain potential therapeutic targets, and the results are presented in the form of a Venn diagram and a table. Cytoscape 3.6.1 software was used to construct the interaction relationship between drugs and potential therapeutic targets, and the results are presented in the form of a network diagram. Then, the STRING (string-db.org/) platform was used to construct the protein-protein interaction (PPI) network of potential therapeutic targets, and the minimum interaction threshold was set as the highest confidence (>0.9) to show the interactions between potential therapeutic targets. Finally, the cytoHubba plugin of Cytoscape 3.6.1 was used to calculate the correlation frequency of potential therapeutic targets, and the results are presented in the form of a network diagram. Then, gene ontology (GO) function and Kyoto Encyclopedia of Genes and Genomes (KEGG) pathway enrichment analyses were performed on the potential therapeutic targets to obtain the gene functions and molecular pathways of the potential therapeutic targets, and the results are presented in the form of bar and dot charts.

### 2.8. Statistical Analysis

SPSS 19.0 software was used to calculate the mean and standard deviation $\left(\bar{\chi }\pm s\right)$ of each group of data, and the differences between each index were compared by ANOVA, with *P* < 0.05 and *P* < 0.01 as the statistical standards. The greyscale values of the protein and nucleic acid images were analysed by ImageJ software, and semiquantitative analysis was carried out. Differences of more than 2-fold were considered significant.

## 3. Results

### 3.1. Inhibitory Effect of SGHZF on Mice with Breast Cancer

#### 3.1.1. Antitumour Effect of SGHZF on Tumour-Bearing Mice

**Figure 1:**
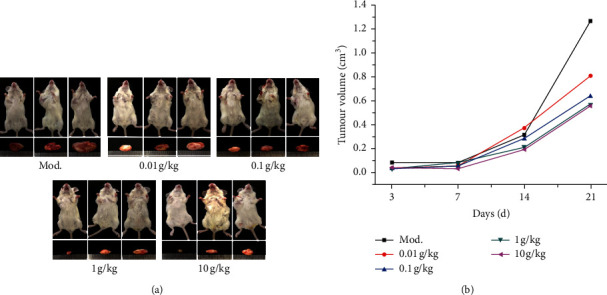
Antitumour effect of SGHZF on tumour-bearing mice. The tumour volume of each mouse was measured and calculated on days 3, 7, 14, and 21, and the inhibition rate was calculated (*n* = 8). On day 21, mice in each group were sacrificed, tumour tissues were harvested, and photographs were taken. (a) Comparison of tumour size among groups on the 21^st^ day. Three typical pictures in each group are shown. (b) Changes in tumour volume.

To verify the inhibitory effect of SGHZF on TNBC, we first conducted tumour inhibition experiments on a mouse model of breast cancer and observed and measured breast cancer tumour growth in each group of mice. The results showed that tumour growth of mice in the model control group was rapid on day 3, while the tumour growth of mice in each experimental group was slow, with no significant difference. The tumour growth of mice in all experimental groups was slow on day 7, while the tumours of mice in the model control group and the 10 g/kg experimental group did not grow significantly. The tumour growth of mice in the model control group was rapid on days 14 and 21, while the tumour growth of mice in each experimental group was significantly inhibited, with a certain concentration dependence observed. Among these observations, the inhibition effect was the most obvious on day 21 in the 1 g/kg and 10 g/kg groups (see Figure 1). The above results indicated that SGHZF inhibits tumour growth in tumour-bearing mice.

#### 3.1.2. Effect of SGHZF on the Expression of c-Myc in Tumour-Bearing Mice

**Figure 2:**
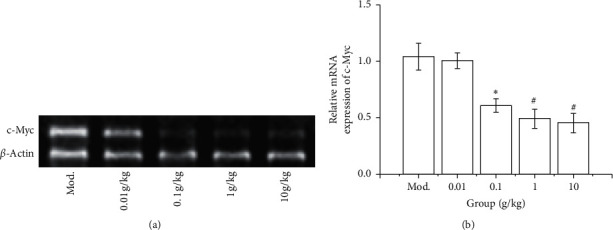
Effect of SGHZF on c-Myc expression in mice with breast cancer. (a) PCR gel map of tumour tissues of mice in each group. (b) Histogram of c-Myc expression in tumour tissues of mice in each group. ^*∗*^Compared with Mod, *P* < 0.05; ^#^compared with Mod, *P* < 0.01.

To further verify the inhibitory effect of SGHZF on TNBC, we used PCR technology to detect the expression of the oncogene c-Myc in the tumour tissues of mice in each group. The results showed that the expression of c-Myc in the 0.1 g/kg, 1 g/kg, and 10 g/kg experimental groups was significantly lower than that in the model control group (*P* < 0.05 or *P* < 0.01). However, there was no significant difference between the 1 g/kg and 10 g/kg experimental groups. The 0.01 g/kg experimental group showed no significant decrease compared with the model control group (see Figure 2). The above results indicated that SGHZF inhibits the expression of the oncogene c-Myc in tumour-bearing mice.

### 3.2. Active Components and Targets of Drugs in SGHZF

**Figure 3:**
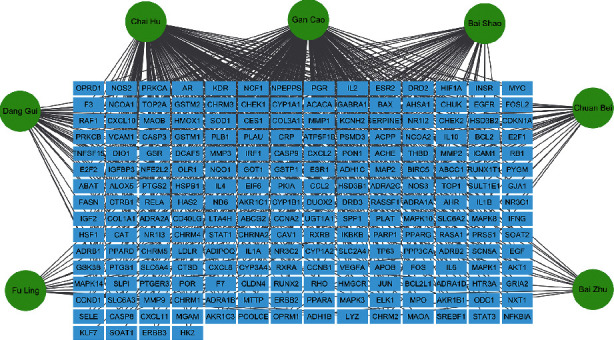
The Chinese herbal medicine-target network. The blue squares represent targets, and the green circles represent the names of the medicines in SGHZF. The line represents the relationship between the active components and the targets.

To obtain the active components and targets of SGHZF, we searched according to the search terms and screening conditions in 2.6 and combined and deduplicated the search results. The results showed that the active components and quantity of the drugs in SGHZF were as follows: 15 species of Chai Hu, 18 species of Bai Shao, 4 species of Bai Zhu, 11 species of Chuan Bei, 6 species of Dang Gui, 7 species of Fu Ling, and 133 species of Gan Cao. After combination and deduplication, 174 active components were obtained. The targets of these 174 active components were further searched, and a total of 214 targets were obtained after combination and deduplication (see Figure 3).

### 3.3. Analysis of the Interaction between SGHZF and TNBC Targets

**Figure 4:**
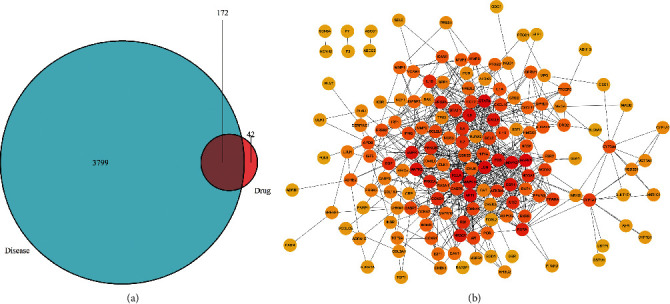
Acquisition and analysis of potential therapeutic targets. (a) Venn diagram of the interaction between the target of drugs in SGHZF and the target of TNBC disease. The blue circle represents the number of targets. The red circle represents the number of drug targets, and the intersection represents the number of potential therapeutic targets. (b) PPI network map with associated potential therapeutic targets. The circle represents the abbreviation of the target, while the line indicates the association between the targets, and the darker the colour, the higher the correlation.

To clarify the interaction relationship between SGHZF and targets of TNBC, we first searched according to the search terms of section 2.6, and then we combined the search results and removed the duplicates. In total, 3606 disease targets were retrieved from the GENECARDS database, and 462 disease targets were retrieved from the OMIM database. After the results of the two databases were combined and duplicates were removed, a total of 3971 TNBC targets were obtained. Then, potential therapeutic targets were obtained by overlapping the SGHZF-related and TNBC-related targets. Ultimately, 172 potential therapeutic targets were obtained through interaction analysis, accounting for more than 80% of TNBC-related targets (Figure 4(a)). Further PPI analysis of potential therapeutic targets revealed that 164 of them were correlated with each other (Figure 4(b)). Among them, highly correlated potential therapeutic targets such as EGFR, ESR1, AKT1, STAT3, and IL6 also showed high disease-related scores in the GENECARDS database (Table 1), which indicated that these potential therapeutic targets may have greater therapeutic effects.

### 3.4. GO Function and KEGG Pathway Enrichment Analysis of Potential Therapeutic Targets

**Figure 5:**
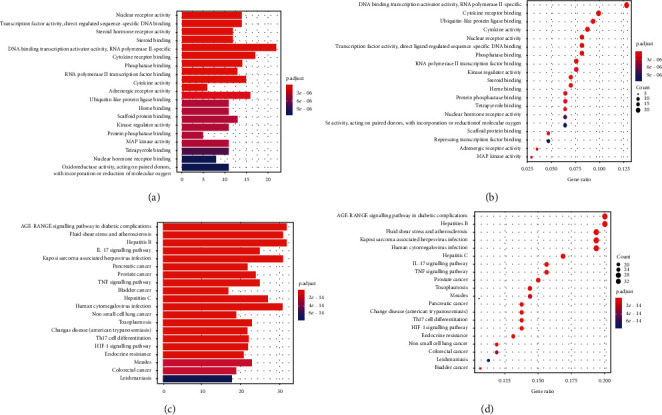
Enrichment analysis of potential therapeutic targets. (a) Bar chart of GO enrichment analysis results; (b) dot chart of GO enrichment analysis results; (c) bar chart of KEGG enrichment analysis results; and (d) dot chart of KEGG enrichment analysis results. In the bar chart, the abscissa represents the number of targets, and the ordinate represents the name of the enrichment result. The redder the colour, the smaller the adjusted *P* value. The bluer the colour, the larger the adjusted *P* value. In the dot chart, the abscissa represents the ratio of targets, and the ordinate represents the name of the enrichment result. The larger the circle, the greater the enrichment and vice versa. The redder the circle, the smaller the adjusted *P* value. The bluer the circle, the larger the adjusted *P* value.

To further investigate the molecular mechanism of potential therapeutic targets, we applied the GO function and KEGG enrichment analysis of potential therapeutic targets. The results of GO functional enrichment showed that the molecular functions of potential therapeutic targets mainly include DNA-binding transcription activator activity, RNA polymerase II-specific activity, and cytokine receptor activity (Figures 5(a) and 5(b)). The results of KEGG enrichment analysis showed that the signalling pathways related to potential therapeutic targets (excluding other disease pathways) mainly included the IL-17 signalling pathway, TNF signalling pathway, and HIF-1 signalling pathway (Figures 5(c) and 5(d)). The above results indicate that the treatment of TNBC with SGHZF is closely related to multiple targets and multiple signalling pathways.

### 3.5. Gene Expression Analysis of Related Targets

#### 3.5.1. Effect of SGHZF on Akt Expression in Mice with Breast Cancer

**Figure 6:**
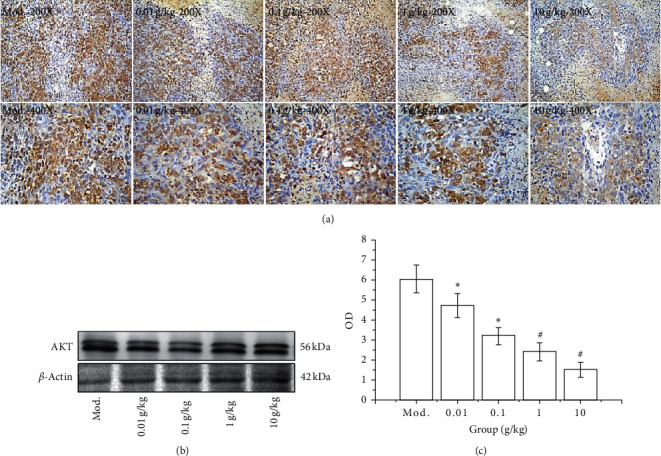
Effect of SGHZF on the expression of AKT in mice with breast cancer. (a) Immunohistochemistry images at 200x and 400x. (b) Gel map of the Western blotting results of each group. (c) OD value of the Western blot in each group. ^*∗*^Compared with Mod, *P* < 0.05; ^#^compared with Mod, *P* < 0.01.

To prove the therapeutic mechanism of SGHZF in mice with breast cancer, we investigated the expression of Akt in the tumour tissue of mice by immunohistochemistry and Western blot analyses. The immunohistochemistry results showed that the tumour tissue of the model control group was dark colour, the cytoplasm was brown-yellow, and the nucleus was light blue, which indicated that the expression of protein molecules was high. In each experimental group, the tumour tissue staining gradually reduced, the brown colour of the cytoplasm gradually weakened, and the blue colour of the nucleus gradually deepened, which indicated that the expression of protein molecules was gradually reduced. The optical density values of immune tissues in each group were Mod > 0.01 g/kg > 0.1 g/kg > 1 g/kg > 10 g/kg. Compared with the values of the model control group, the values of each experimental group were significantly different (*P* < 0.05 or *P* < 0.01), and the differences were most obvious in the 1 g/kg and 10 g/kg groups (see Figure 6(a)). The Western blotting results showed that the expression of AKT in each group showed a downward trend with a significant concentration dependence (*P* < 0.05 or *P* < 0.01), which was consistent with the immunohistochemical results (see Figures 6(b) and 6(c)). The above results indicate that SGHZF inhibits the expression of Akt in mice with breast cancer.

#### 3.5.2. Effect of SGHZF on HIF-1*α* Expression in Mice with Breast Cancer

**Figure 7:**
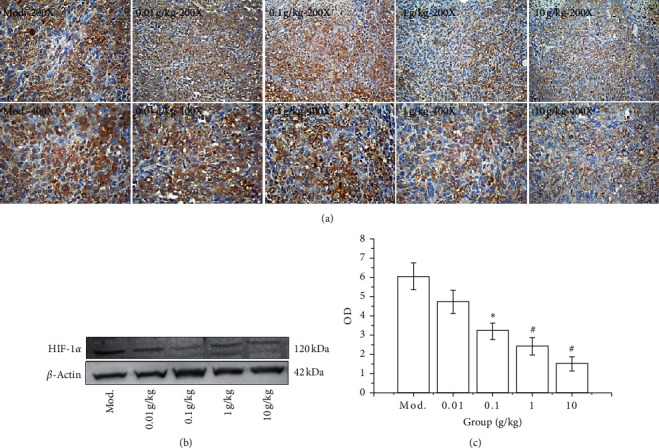
Effect of SGHZF on the expression of HIF-1*α* in mice with breast cancer. (a) Immunohistochemistry images at 200x and 400x. (b) Gel map of the Western blotting results of each group. (c) OD value of the Western blot in each group. ^*∗*^Compared with Mod, *P* < 0.05; ^*∗*^compared with Mod, *P* < 0.01.

To prove the therapeutic mechanism of SGHZF in mice with breast cancer, we further investigated the expression of HIF-1*α* in the tumour tissue of mice by immunohistochemistry and Western blot analyses. The immunohistochemistry results showed that the tumour tissue of each group was brown yellow, while the colour of the model control group was darker, and the distribution was uniform. In each experimental group, the tumour tissue was light coloured; the brown-yellow staining of the cytoplasm was gradually reduced, but the light blue staining of the nuclei gradually deepened, making the whole tissue appear as light blue-white. The optical density values of the immune tissues in each group were ranked Mod >0.01 g/kg > 0.1 g/kg > 1 g/kg > 10 g/kg, which showed an obvious concentration dependence. The difference between the values of the model control group and the 0.1 g/kg experimental group was significant (*P* < 0.05); the difference between the values of the 1 g/kg and 10 g/kg experimental groups was extremely significant (*P* < 0.01) (see Figure 7(a)). The Western blotting results showed that the expression of HIF-1*α* in each group showed a downward trend with a significant concentration dependence (*P* < 0.05 or *P* < 0.01), which was consistent with the immunohistochemical results (see Figures 7(b) and 7(c)). The above results indicate that SGHZF has an inhibitory effect on the expression of HIF-1*α* in mice with breast cancer.

## 4. Discussion

TNBC is a kind of heterogeneous tumour. Compared with other molecular types of breast cancer, it has a higher recurrence rate and a poorer prognosis [15, 16]. At present, chemotherapy is still the main treatment for TNBC. Despite aggressive systemic chemotherapy, the survival of patients with TNBC is unacceptable [17, 18], and this is exemplified in older patients [19]; chemotherapy also leads to inevitable drug resistance and side effects [20]. Although scholars have continued their search for druggable molecular targets in TNBC, to date, no specific drug has been found to be effective [21]. A large number of studies have shown that the treatment of cancer with traditional Chinese herbal formulas involves the interaction of multiple targets and multiple signalling pathways [22–24]. Therefore, TCM may have great potential for the treatment of TNBC.

Considering that the growth of tumours can be observed directly in animal experiments in vivo and that tumour tissues can be assessed more conveniently, we used the mouse 4T1 breast cancer animal model to study the inhibitory effect of SGHZF on TNBC. This model is a recognized animal model of TNBC in mice, and its tumour growth rate and metastasis status are very similar to those of humans, which makes it an ideal animal model for the study of TNBC [25, 26]. In this study, we calculated the tumour volume of mice in this model and found that the tumour volume and tumour inhibition rate of different experimental groups did not change over time. However, on day 21, there were statistically significant differences in the tumour volumes and tumour inhibition rates between groups, which may be related to the metabolism and blood drug concentration of the traditional Chinese herbal formula in mice. Our experiment confirmed that SGHZF inhibits tumour growth in tumour-bearing mice.

As an important member of the Myc family, c-Myc is an oncogene in breast cancer [27]. It plays an important role in promoting the occurrence and development of tumours [28] and in promoting and maintaining tumour cell proliferation [29], differentiation, and apoptosis [30]. In recent years, a large number of studies have shown that c-Myc plays an important role in regulating the metabolism, proliferation, and apoptosis of TNBC cells [30–32]. Therefore, we measured the expression of c-Myc in tumour tissue. The results showed that the expression of c-Myc in the high-dose SGHZF experimental group was significantly reduced compared to that in the control group, which indicates that SGHZF had an inhibitory effect on c-Myc in mouse breast cancer.

After confirming that SGHZF has an inhibitory effect on breast cancer in tumour-bearing mice, we investigated the active components and targets of drugs in SGHZF. SGHZF contains a large number of traditional Chinese herbs, and its active components and molecular functions are complex and diverse, so it is difficult for traditional research methods to comprehensively study the molecular mechanism of its treatment. Therefore, we applied the network pharmacology research method to explore the potential therapeutic targets of SGHZF. Through network pharmacology research, we obtained 174 active components and 214 targets in SGHZF. The active components and targets of Chai Hu and Gan Cao are relatively high, indicating that these two drugs may be the main drugs in SGHZF. According to the literature, glycyrrhizin and glycyrrhetinic acid combined with etoposide could inhibit proliferation and promote apoptosis of TNBC cells [33]. Saikosaponin *D* from Chai Hu suppresses TNBC cell growth by targeting *β*-catenin signalling [34].

Through further study, we found that there were 172 potential therapeutic targets of SGHZF, accounting for more than 80% of the targets of SGHZF, among which 164 potential therapeutic targets were correlated with each other. By comparing the correlation scores of disease targets, we found that the potential therapeutic targets with high PPI correlations, such as EGFR, ESR1, AKT1, STAT3, and IL6, also showed high disease correlation scores in the GENECARDS database, indicating that these targets are closely related to TNBC and suggesting that their potential therapeutic effects may be greater. The results of GO functional enrichment analysis showed that the molecular functions of potential therapeutic targets mainly included DNA-binding transcription activator activity, RNA polymerase II-specific activity, and cytokine receptor activity. These molecular functions and characteristics are closely related to tumour cells. The results of the KEGG pathway enrichment analysis showed that the pathways related to potential therapeutic targets (excluding other disease pathways) mainly included the IL-17 signalling pathway, the TNF signalling pathway, and the HIF-1 signalling pathway, which have been associated with tumours [35–37].

Among the enriched KEGG pathways, the HIF-1 signalling pathway is most closely related to TNBC [38]. An increase in HIF-1 expression is one of the main molecular characteristics of TNBC [39]. HIF-1*α* is the main target of the HIF-1 signalling pathway [40], and the overactivation of Akt upstream can activate the expression of HIF-1*α* downstream [41]. A large number of studies have shown that hypoxia can enhance the invasive ability of cancer [42], which is closely related to chemotherapeutic resistance [43]. Considering that TNBC is highly invasive and is mainly treated with chemotherapy, we believe that the HIF-1 signalling pathway is very important to TNBC. Moreover, the PPI analysis revealed that HIF-1*α* and AKT1 of the Akt family were highly correlated with potential therapeutic targets. Therefore, we chose Akt and HIF-1*α* for further experimental verification. The results indicated that SGHZF can inhibit the expression of AKT and HIF-1*α*, which is consistent with the network pharmacology prediction. Through this study, we found that SGHZF has an inhibitory effect on mouse TNBC and that its mechanism may be related to the inhibition of Akt and HIF-1*α* expression. Thus far, we have preliminarily explored the molecular mechanism of SGHZF in the treatment of TNBC.

In summary, as a complex Chinese herbal compound, SGHZF has a multifaceted molecular mechanism in the treatment of TNBC. In this study, we preliminarily identified and verified this mechanism, which lays a foundation for the follow-up study of SGHZF in the treatment of TNBC.

## 5. Conclusions

SGHZF inhibits breast cancer tumour growth in mice, and the underlying mechanism may be related to the inhibition of Akt and HIF-1*α* expression.

## Figures and Tables

**Table 1:** Potential therapeutic targets with high PPI correlation and high disease correlation scores in the GENECARDS database.

Target	Interactions in the PPI network	Disease-related scores in the GENECARDS database
EGFR	22	104.07
ESR1	21	102.25
AKT1	36	82.32
STAT3	38	70.64
IL6	26	67.01

## Data Availability

The data used to support the findings of this study are available from the corresponding author upon request.
